# Bias and comparison framework for abusive language datasets

**DOI:** 10.1007/s43681-021-00081-0

**Published:** 2021-07-19

**Authors:** Maximilian Wich, Tobias Eder, Hala Al Kuwatly, Georg Groh

**Affiliations:** Technical University of Munich, Munich, Germany

**Keywords:** Hate speech detection, Abusive language detection, English, Arabic, Bias

## Abstract

Recently, numerous datasets have been produced as research activities in the field of automatic detection of abusive language or hate speech have increased. A problem with this diversity is that they often differ, among other things, in context, platform, sampling process, collection strategy, and labeling schema. There have been surveys on these datasets, but they compare the datasets only superficially. Therefore, we developed a bias and comparison framework for abusive language datasets for their in-depth analysis and to provide a comparison of five English and six Arabic datasets. We make this framework available to researchers and data scientists who work with such datasets to be aware of the properties of the datasets and consider them in their work.

## Introduction

The last few years have seen an increase in popularity for abusive language detection as a classification problem. This growing interest brought along the release of a more significant number of labeled datasets. Although this increase in the available data has made research more accessible, no real benchmark dataset for abusive language detection has been established with a unique set of problems in the domain, chiefly encompassing comparability issues between systems trained and evaluated on various datasets [25, 38]. These problems emerge from differences between the datasets, such as context, platform, sampling process, and labeling, with even the task definition being subtly different in many cases [14].

A further aspect that impairs the dataset comparability is biased data. We define bias as a phenomenon in which a system “systematically and unfairly discriminate[s] against certain individuals or groups of individuals in favor of others” [17, p. 332]. In the context of abusive language, bias can be materialized in different forms. One example is topic bias [47]. Let us assume that we have an abusive language dataset with a neutral and an abusive class. If the abusive class is dominated by a particular topic that is not abusive per se (e.g., sports) and the neutral class does not contain many documents about this topic, a classification model learns to use terms from this topic to distinguish between both classes [47]. Consequently, the classifier systematically discriminates documents related to this topic. Therefore, it is necessary to uncover bias in datasets and make them transparent.

Recently, frameworks for documenting datasets characteristics have been proposed, such as [18] and [5]. Transparency in the processes to create new datasets can be realized using the guidelines outlined in these frameworks, but they do not solve the problem for existing datasets and comparisons beyond the mostly discreet metrics within specific tasks. Even where such information is available for a single dataset, it is hard to quantify how differences in data collection or labeling choices manifest themselves in the systems built on top of them. In the worst case, this blind spot can lead to strongly biased systems, which inherit some of the systemic problems stemming from the underlying data without this becoming evident from evaluation according to common metrics. Consequently, the further use of these systems is also highly problematic from an ethical standpoint. Without insight into the training data’s actual properties and distribution, there are no guarantees that the system performs fairly in a real-world setting.

Therefore, the paper aims to provide a framework to compare abusive language datasets and uncover their inherent properties (e.g., different forms of bias). Furthermore, we can help the research community bring order and structure to the variety of abusive language datasets by providing two main contributions:

1. A structured framework for analyzing and comparing abusive language datasets across various fine-grained metrics. The chosen metrics apply across multiple dimensions, capturing meta-information, semantic information, annotations, and derivative measures based on state-of-the-art classification evaluated on these datasets. The framework is not limited to the English or Arabic language. It can also be applied to datasets in other languages.
2. The paper provides an excellent comparison of five English and six Arabic datasets from the abusive language domain, which illustrates their differences, highlights their focus, and reveals potential biases and hidden properties.

The paper is structured as follows: Sect. 2 discusses related work and explains why our work fills a demand that other researchers have not covered. In Sect. 3, we describe our developed framework and outline the reasons for adding the various methods. Afterward, we introduce our data selection for the two case studies: (1) English and (2) Arabic datasets. The results of the case studies are presented in Sects. 5 and 6. Section 7 contains a discussion about our findings and challenges of current and prospective abusive language datasets. Finally, in Sect. 8, we conclude our work.

## Related work

The growing number of abusive language datasets has led to a range of dataset surveys in recent years. However, most of the early research on hate speech or abusive language data was done as part of an overview of the emerging field’s methodology, including reviews such as [14, 38], and [24] discussing key properties of a few selected datasets used in existing systems.

A more comprehensive study on abusive language datasets was published by [39], compiling 51 datasets. They proposed a more involved descriptive framework, including information on the target of abuse, the level of annotation, and the class distribution. Further surveys followed, including [32] on 49 datasets, which was the first to include a short specific lexical analysis of the dataset contents to identify topic bias. [25] has recently given an overview of 17 datasets to evaluate them on their ability to function as benchmark datasets in the abusive language domain, assessing aspects such as availability, class imbalance, exact task definition, and label conflation. All dataset surveys have in common that they conduct a high-level comparison (e.g., number of documents, source, and data collection strategy) and do not look beyond the surface except the limited lexical analysis in [32]. Consequently, these surveys are satisfactory for identifying in broad strokes how different datasets compare on an annotation level. However, they do not provide details of the dataset contents, as they rely mainly on second-order descriptions about the data, principally compiled for the release of a specific dataset.

[18] and [5] proposed datasheets for datasets to document their characteristics because the machine learning or NLP communities do not have a standardized approach. These data sheets are necessary and make it easier to compare datasets. However, they cannot be applied to already published datasets, and in-depth analysis and comparison of different data sets are not possible. The type of work required is necessary to be done by the original authors of the dataset. Furthermore, the only recourse for a practitioner trying to work with a dataset for which no datasheet was released would be to contact the original authors and ask about the specific creation information. Furthermore, they do not reflect all characteristics of abusive language datasets, being a very general framework for all data types.

A range of other research has dealt with evaluating specific datasets or systems to uncover bias problems with the underlying data. [30] evaluated gender bias on the [44] and [16] datasets. [13] investigated unintended bias with respect to identify terms and proposed a method to debias the training data. [9] reported problems with the association of minority group language with hate in their data, while [47] have done work on the influence of different biases in the sampling of popular abusive language datasets (e.g., topic and author bias). [46] analyzed how political bias influence hate speech classification models. [37] proposed social bias frames, which is a formalism that “aims to model the pragmatic frames in which people project social biases and stereotypes onto others” [37, p. 1]. Another form of bias in abusive language datasets that researchers have addressed is annotator bias. [36] investigated annotator bias concerning the Afro-American English (AAE) dialect. They showed that a classifier trained on a standard abusive language dataset discriminates documents in AAE. [1] identified annotator bias by splitting annotators according to their demographic characteristics. The challenge of this approach is that it requires demographic data of the annotators. [45] addressed this problem by identifying annotator bias purely on similarities in the annotation behavior. Lastly, [15] compared six popular datasets according to their differences in class labeling via similarity in a common word embedding space and further classifying them with the Perspective API framework.

To the best of our knowledge, no one has developed a framework to conduct an in-depth comparison of abusive language datasets focusing on various forms of bias.

## Framework

**Fig. 1:**
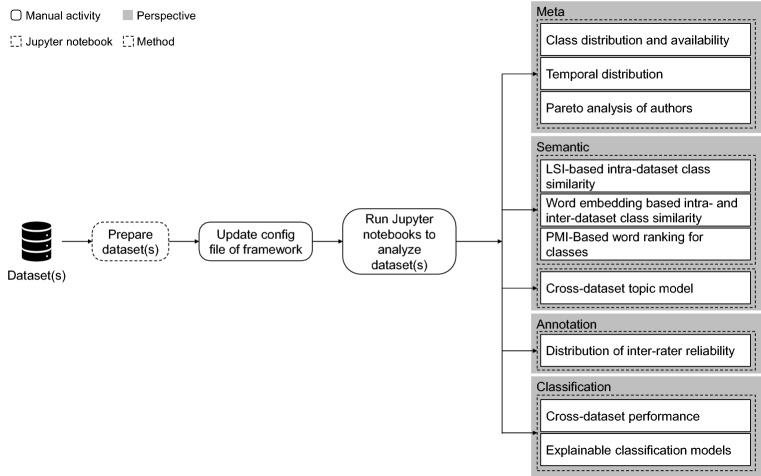
Overview of the framework’s methods and the required data

Since there is a need for systematical in-depth analysis and comparison of abusive language datasets going beyond high-level properties, we propose the following framework. It consists of three perspectives that contain methods addressing various challenges. Table 1 provides an overview of the framework and the challenges that are addressed by the methods. Figure 1 displays the steps how the framework is applied. We also published our code^1^ to encourage researchers to use the framework for their research. The framework is a collection of Python scripts and modular Jupyter notebooks that contain the individual parts of the analysis and use a unified framework to handle data input for all parts of the analysis.

**Table 1:** Bias framework for abusive language datasets

Perspective	Method	Problem
1. Meta	(a) Class distribution and availability	Degradation
	(b) Time distribution	Temporal bias
	(c) Pareto analysis of authors	Author bias
2. Semantic	(a) LSI-based intra-dataset class similarity	Similarity/dissimilarity of classes
	(b) Word embedding based intra- and inter-dataset class similarity	Similarity/dissimilarity of classes
	(c) Cross-dataset topic model	Topic bias
	(d) PMI-Based word ranking for class	Topic bias
3. Annotation	(a) Distribution of inter-rater reliability	Annotator bias
4. Classification	(a) Cross-dataset performance	Generalizability
	(b) Explainable classification models	Generalizability

### Meta perspective

The first perspective focuses on the metadata of the documents within the datasets. It leaves the textual data out and emphasizes aspects, such as class distribution or author distribution.

#### Class distribution and availability

The first method investigates the class distribution and availability of data. We do this since some datasets, especially those collected from Twitter, often contain only references to the documents (e.g., tweet IDs) due to platform policies’ restrictions. Sharing only references is problematic because documents on online platforms can be deleted over time [41]. Particularly, documents with hateful or abusive content are prone to removal since they often violate the platform policies. This degradation impairs both the quality and quantity of datasets. To avoid degradation, some researchers publish datasets containing the text—sometimes anonymized (e.g., removing usernames from the documents)—instead of a reference to the original resource. It solves the degradation issue but exacerbates other data analyses (e.g., temporal or author distribution). Therefore, we include the analysis of class distribution and availability in our framework.

#### Temporal distribution

Another challenge of abusive language detection is evolving language [14, 33, 41]. Words and expressions that are unproblematic today might have an abusive connotation tomorrow. Consequently, a classification model trained on an older dataset can perform worse on new datasets because the model does not recognize new language patterns [41].

If the collected data was created quickly (e.g., only in a few weeks), it can indicate that the abusive data contains only current abusive language patterns and covers only current topics (e.g., refugee crisis). As a result, classification models trained on such a dataset might perform worse on other datasets from other periods or with another topical focus. Thus, it is interesting to investigate when the documents were created and to identify a temporal bias.

#### Author distribution

An aspect that is also of interest is whether the dataset has an author bias [47]. That means that a small number of users created a large portion of documents from one or more classes. The problem of author bias is that a classification model trained on such a dataset tends to memorize the author’s writing style or the topics they are writing about but not actual indicators of hateful language [47]. Therefore, we propose a Pareto analysis of the authors combined with a class distribution to make this transparent and uncover author bias. It is a method based on quality management and supports root cause analysis [43]. In our case, we count the number of documents for each user and rank them according to the number of documents. Consequently, we can figure out whether a large portion of documents is produced by a small number of authors, signifying author bias.

### Semantic perspective

After investigating the metadata, we focus on the semantic level of the datasets. Then we analyze the class similarities within and across the datasets and the topics addressed by them.

#### LSI-based intra-dataset class similarity

Before explaining the method, we explain why class similarity is relevant to our framework. Firstly, the more similar documents within a class are, the easier it is for a classification model to distinguish it from other classes. Secondly, the more dissimilar the two classes, the easier it is for a classifier to distinguish between both. Therefore, similarity scores can also act as an indicator of the classifier’s generalizability.

The first method focuses on the inter- and intra-class similarities within a dataset. It applies Latent Semantic Indexing (LSI) [11] and uses cosine distance to compute the similarity within a class and between the classes because it does not require any pre-trained word embeddings, and we do not want to rely on word embedding based methods in this perspective. The result is a matrix for each dataset, representing the homogeneity and similarity of the dataset’s classes. The findings are comparable between different datasets, but they do not demonstrate the similarity of different datasets classes, which is addressed by the following method.

#### Word embedding based inter- and intra-dataset class similarity

In order to compare the class similarities across the datasets, we apply a variant of the method proposed by [15]. After preprocessing (e.g., removing URLs, usernames), we use a pre-trained FASTTEXT embedding depending on the dataset’s language to compute a document vector for each document [26]. This step is slightly different from Fortuna et al.’s methods [15]. Instead of averaging the word vectors of a document to get the document vector, we use FASTTEXT’s sentence embedding feature. Then, all word vectors of a class are averaged, obtaining a centroid for the class. In the last step, Principle Component Analysis (PCA) [31] is applied to compute a 2-dimensional representation, visualizing the similarity between the classes across the datasets, as proposed by [15].

#### PMI-based word ranking for classes

The third method of the semantic perspective produces a listing of the most relevant terms for each class of a dataset proposed by [47]. The intention is to provide an impression of what the class is about and what classification models learn. In order to calculate the relevance of the terms, we use pointwise mutual information (PMI) [8]. However, instead of computing the PMI between two words, our pair consists of the word $w_i$ and class $c_j$:

1 $$\begin{aligned} pmi(w_i,c_j) = log\frac{p(w_i,c_j)}{p(w_i)p(c_j)} \end{aligned}$$

As a result, we obtain a value representing the relevance of the word for the class. We can identify the most relevant terms by ranking them. However, this method does not describe the class to its full extent but is a good indication.

#### Overarching topic modeling

The fourth method of the semantic perspective analyzes topic bias in datasets. It is often caused by the way how the datasets are collected. Some abusive language datasets, for example, are gathered through a keyword-based approach (e.g., hashtag-based filtering of tweets). But if the keywords are too specific, the resulting dataset can exhibit topic bias. The focus on topic bias is motivated as follows: If an entire dataset focuses on one or a few specific topics, the model’s generalizability is impaired, meaning it performs poorly on other datasets. Let us assume that we have an abusive language dataset that mainly contains COVID-19-related content. A model trained on the dataset might perform worse on an abusive language dataset with a sports focus. Therefore, it is necessary to identify topic bias in a dataset. We suggest the following topic model-based method to investigate this phenomenon.

In the first step, we sample *n* documents from each of the *m* datasets to be analyzed and merge into one dataset. Two different sampling strategies are proposed: (1) sampling according to the actual class distribution, (2) sampling an equal number from each class. The first one delivers a more representative result, while the second gives more weight to underrepresented classes. In the second step, we use CluWords to generate a topic model of the merged dataset with *l* topics. CluWords is a topic model algorithm that uses word embeddings and non-probabilistic matrix factorization and works well on short texts [42]. As word embedding, we use the same FASTTEXT model as in the previous method. Besides the *l* topics, CluWords outputs outputs a one-dimensional vector for each document, signifying the document’s topic distribution. Additionally, we generate for each topic a one-dimensional vector as representation. In the third step, we apply t-SNE to project the *l*-dimensional vectors of the documents and the topic centroids to a two-dimensional representation [23]. After coloring each document data point depending on its dataset, we can use the plot to visualize the topic distribution and uncover topic bias.

### Annotation perspective

The third perspective deals with the annotations of the data provided by humans. As studies have shown [1, 36, 37, 45], biased annotations can impair classification performance. Consequently, it must be addressed by our framework.

#### Distribution of inter-rater reliability

We recommend examining the distribution of the annotator’s inter-rater reliability to uncover potential annotator bias. Low inter-rater reliability implies “systematically biased coders” [35, p. 673]. Therefore, we suggest analyzing the overall inter-rater reliability of a dataset and the individual inter-rater reliability of each annotator. The overall metric indicates the quality of the annotations and a potential annotations bias. Moreover, the individual metrics help us understand whether a general disagreement between the annotators causes low inter-rater reliability or a few strongly biased annotators. Krippendorff’s alpha is utilized as an inter-rater reliability metric because it can handle missing annotations where each annotator’s vote is required to conduct the analysis [19]. However, most datasets only provide an aggregated gold standard, making it impossible to apply this method.

### Classification perspective

The fourth perspective compares and investigates the classification models separately trained on the different datasets and evaluated on all test sets. The goal is to assess the generalizability and to identify blind spots of the underlying datasets.

#### Cross-dataset performance

The goal of abusive language detection research is to build classification models that reliably detect abusive language. One key component to reach this is training data that covers the diversity and multifacetedness of abusive language. Using such data, we can build more generalizable models. This aspect is related to bias in a dataset: the more significant and stronger the bias is in a dataset, the less generalizable its trained model. Therefore, we integrate it into our framework and propose the following method to compare the generalizability of datasets.

We train a classifier for each dataset and test it on the test sets of the other datasets. Firstly, we sample an equal number of documents from each dataset and split them into training and test set (80:20). Identical training and test set sizes are necessary to receive comparable results. Subsequently, we merge the classes so that we get a binary task (*neutral* and *abusive*). It is necessary because there is no standard labeling schema for abusive language. Most datasets can be converted to binary tasks. After preprocessing the documents, we train classification models for each dataset. For the classifier, we fine-tune a pre-trained BERT model depending on the language of the datasets for our task. After training the classifiers, we evaluate them on all test sets and a combined test set that consists of equal samples of documents from all tests. The results show how well a classifier trained on one dataset performs on unfamiliar datasets, demonstrating the generalizability of a classifier and its corresponding dataset.

#### Explainable classification models

The previous method provides a useful overview of the dataset’s generalizability. We recommend a method to analyze the classifiers with an explainable AI technique to study the classifiers and uncover their blind spots or weak points.

Therefore, the models trained by the previous method are combined with the SHAP framework, which provides a set of different methods to explain predictions [22]. Concretely, we apply the Partition SHAP method—a model-agnostic local explainability method that relies on Owen values to explain single predictions [21].

Our method’s outcome is the following: For a given document from the combined test set, we receive a prediction and an explanation for each dataset. The explanation shows how each word contributes to the prediction of the classifier. So, we can compare the different classification models in-depth and identify weak points of the classifiers because we see what is relevant for the classifier and what is not. These insights also help to uncover bias. For example, a classification model classifies a nonabusive document as abusive, and the explanation marks the word *Islam* as highly relevant for the prediction. This can indicate a religious bias in the data, caused by the fact that the word *Islam* occurs more frequently in the abusive class than in the neutral class.

## Data

Our developed framework is meant to be a tool for researchers and data scientists that work with abusive language datasets or create such datasets. In order to demonstrate its usage, we apply the framework to five English and six Arabic datasets listed in Table 2. We selected English because most abusive language resources are written in English, and Arabic because it fundamentally differs from English. In contrast to other dataset reviews, we compare only a small number of datasets due to our comprehensive, in-depth analyses; considering more datasets would go beyond the scope of this article.

Our dataset selection focuses on Twitter as the primary data source to ensure the comparability of the datasets. A further criterion is the size of the dataset. Since we draw even samples from all datasets for some analyses, the smallest dataset has 4,000 tweets.

The first three English datasets are commonly used by the research community [32], Zampieri is from the shared task OffensEval 2019 [48], and Vidgen is a relatively new dataset about COVID-19-related abusive language [40]. The latter was selected because it comprises an entirely different context. Regarding the Arabic datasets, we picked the Twitter datasets that we found and are not too small. One of them is also from a shared task—Mubarak (OSACT4 Arabic Offensive Language Detection Shared Task) [28]. Finally, Chowdhury consists of tweets and comments from Facebook and YouTube, making it an interesting dataset to compare to the others [7].

Some proposed methods (e.g., classification) require a unified labeling schema. Therefore, we convert the labels to a binary labeling schema—*neutral* and *abusive*. The *abusive* class comprises all classes that refer to abusive, offensive, or hateful language. Moreover, the bold-faced classes in Table 2 are those that are labeled as *abusive*.

**Table 2:** Selected abusive language datasets (class names in bold are the abusive categories)

Lang.	Name	Source	Size	Labels	Ref.
English	Waseem	Twitter	16,907	None, sexism, racsim	[44]
	Davidson	Twitter	24,783	Offensive, hate, neither	[10]
	Founta	Twitter	99,996	Normal, abusive, hateful, spam	[16]
	Zampieri	Twitter	14,100	Hierarchical labels: (1) not offensive, offensive (2) if offensive: targeted insult, untargeted insult (3) if targeted: individual target, group target, other	[48]
	Vidgen	Twitter	20,000	Hostility, criticism, counter speech, discussion of East Asian prejudice, neutral	[40]
Arabic	Alsafari	Twitter	5341	3-class: clean, offensive, hate; 6-class: clean, offensive, religious hate, gender hate, nationality hate, ethnicity hate	[3]
	Alshalan	Twitter	8958	Hate, non-hate	[4]
	Albadi	Twitter	6136	Hierarchical labels: (1) neutral, religious hate (2) if religious hate: Muslims, Jews, Christians, Atheists, Sunnis, Shia, other	[2]
	Chowdhury	Twitter, Facebook, YouTube	4000	Hierarchical labels: (1) non-offensive, offensive (2) if offensive: vulgar, hate, only offensive	[7]
	Mubarak	Twitter	9996	Hierarchical labels: (1) non-offensive, offensive (2) if offensive: hate speech, not hate speech	[28]
	Mulki	Twitter	5846	Normal, busive, hate	[29]

## Case study 1- english datasets

### Meta perspective

#### Class distribution and availability

**Fig. 2:**
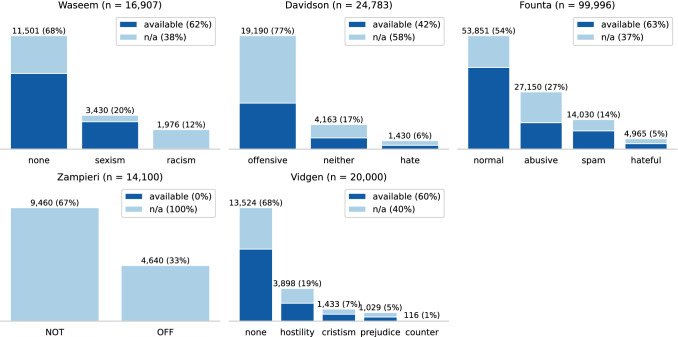
Class distribution and platform availability of English datasets (*available* means that the online resource, e.g. tweet, is still accessible)

Figure 2 presents the class distributions and data available on the social media platform (Twitter) of the English datasets. The number next to the dataset name is the total number of documents in the dataset. The percentage value on top of each bar and in the legend states the class’s relative share and reflects how much of the entire dataset is still accessible online, respectively.

The first observation is that all datasets are imbalanced. In all datasets, except Davidson, the abusive language-related classes are underrepresented. In the case of Davidson, the *offensive* class has a share of 77%, while *neither*’s share is 17%, and *hate*’s is only 6%. In regards to the data availability, we observe a degradation between 37% and 58%. It is not surprising that the hate-related classes (e.g., *racism* of Waseem, *offensive* of Davidson, *abusive* of Founta) are more affected by degradation because these tweets violate Twitter’s community guidelines. The 0% availability of the Zampieri is not representative because we do not know how many tweets are still accessible online due to the missing tweet IDs. This is also why we cannot perform the following two analysis methods on the Zampieri dataset.

#### Temporal distribution

**Fig. 3:**
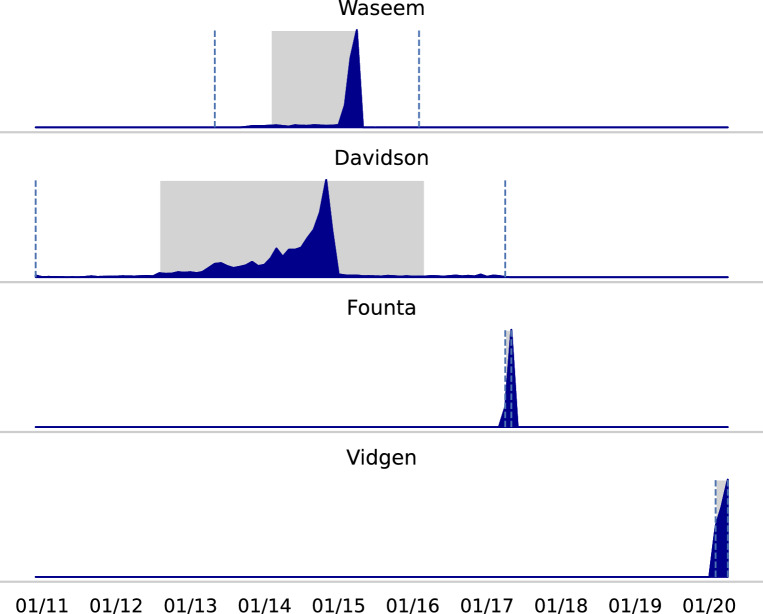
Temporal distribution of the tweets from English datasets with tweet IDs

Figure 3 visualized the distribution when the tweets were posted. The dotted lines represent the timestamps of the first and last tweets, while the gray area marks the 95% percentile.

While all documents from Founta and Vidgen were created in a short period of time, the ones from Waseem and especially the ones from Davidson cover a more extended period. The latter is beneficial for training generalizable classifiers because it comprises linguistic traits from various periods—especially in the context of quickly evolving day-to-day languages. Further observations are that the datasets are from different years and that there are approximately five years between the oldest and newest. If the data is too old, it can have a negative impact because classifiers trained on this data struggle to identify recent abusive language expressions. Therefore, abusive language datasets should be up-to-date.

#### Author distribution

**Fig. 4:**
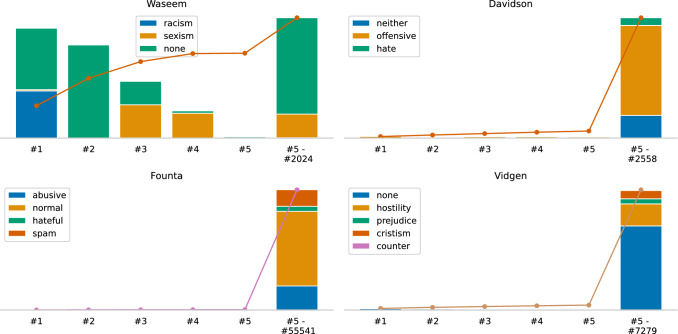
Pareto analysis showing how many tweets (incl. classes) were created by the top authors of each dataset

Figure 4 illustrates the Pareto analysis of the documents’ authors. Due to degradation, the analysis only considers the tweets that are still available on Twitter^2^. We observe that the Waseem dataset has an obvious author bias because nearly all racist tweets were created by one author and a large portion of the sexism tweets by two authors. In contrast, the other datasets do not contain an imbalance with respect to the authors and their tweets.

### Semantic perspective

#### LSI-based intra-dataset class similarity

**Fig. 5:**
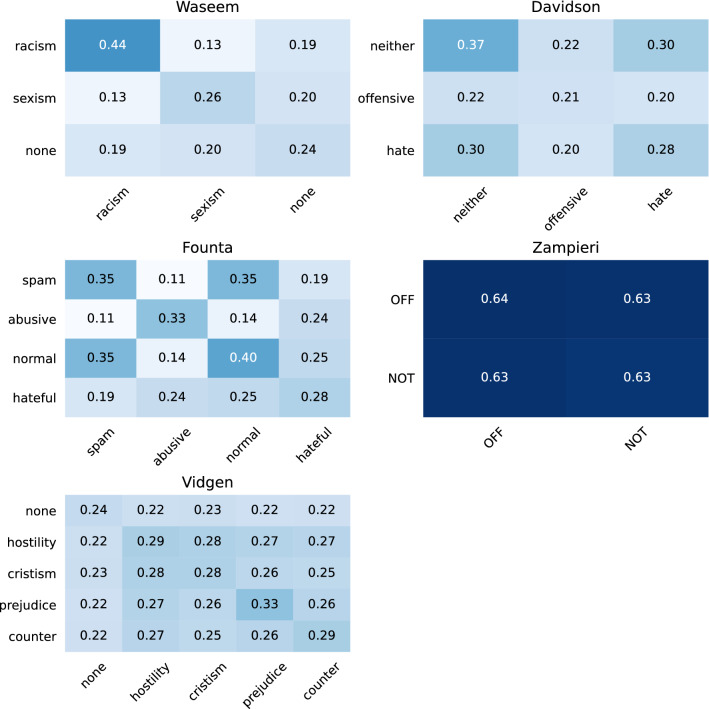
LSI-based similarity of classes within English datasets (the higher the score, the more similar are the two classes

Figure 5 displays the results of the LSI-based intra-dataset class similarity. The scores are between zero and one. The higher the score, the more homogeneous or similar the two classes.

The first observation is that the LSI scores of Zampieri are higher than those of the other datasets. That means that the classes are more homogeneous than those from the other datasets, but both classes are also similar. A contrast to these two classes is the racism and sexism classes of Waseem. They are more homogeneous by themselves than they are similar to each other. Concerning Founta, we see that the spam and normal class are very similar, while the abusive one is distinguishable from these two classes. The hateful class is less homogeneous than the other three and is also similar to the other three. Finally, Vidgen exhibits constant LSI scores both within and between the classes, suggesting a balanced dataset composition.

#### Document embedding based intra- and inter-dataset class similarity

**Fig. 6:**
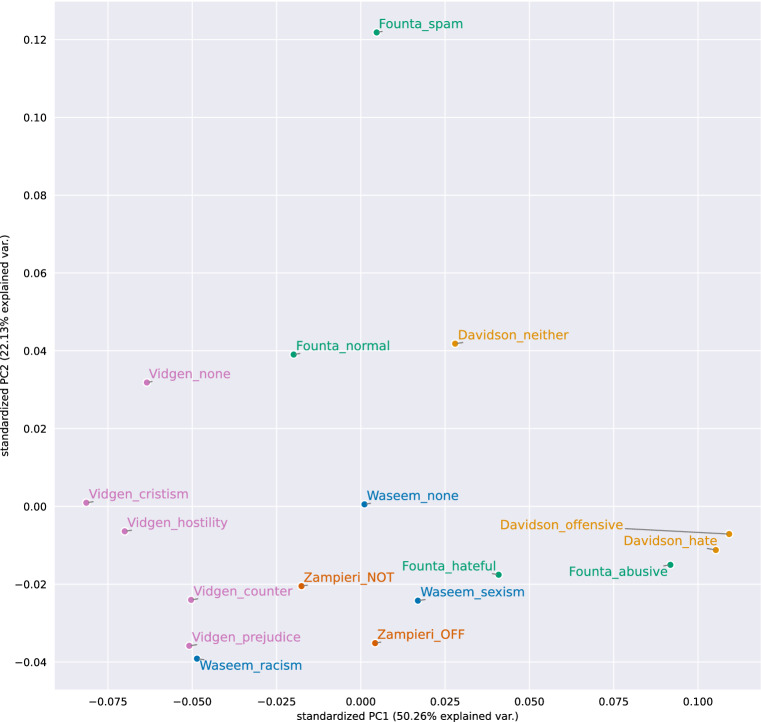
FASTTEXT sentence embedding vectors averaged for each class of English datasets and visualized with PCA (the closer the points, the more similar the classes)

Figure 6 visualizes the similarities between the classes of all datasets based on the averaged FASTTEXT document vectors and PCA. We can observe that each dataset’s classes are approximately grouped, signifying coherence within the dataset. One outlier is the *spam* class of Founta. It was a good decision from the authors of the Founta dataset to introduce a spam class. Otherwise, the documents would have fallen in the normal class, making it easier for classifiers to distinguish between abusive and normal content without actually learning the differences between these classes. Furthermore, the racism class of Waseem and the prejudice from Vidgen seem to be quite similar. As racism often contains prejudice, this similarity should not surprise us. Besides that, Vidgen’s other classes are separated from the rest, which can be traced back to the topical focus of the dataset. Additionally, we can see that some hate-related classes (*sexism* of Waseem, *hate* and *offensive* of Davidson, and *hateful* and *hateful* of Founta) exhibit a certain degree of similarity. We can observe this grouping effect also at the neutral classes of Vidgen, Founta, and Davidson.

#### PMI-based word ranking for classes

Table 3 presents the words with the highest PMI from the abusive classes, demonstrating what the classes represent. It is not surprising that the abusive classes of Davidson and Founta contain many swearwords. Furthermore, we can see that the *racism* class of Waseem focuses on religious topics, especially Islam. Another interesting observation is the dominance of political terms in the Zampieri. Similar to Zampieri, the hostility class of Vidgen steps out the lines. The most relevant phrases are related to viruses and China, which we can trace back to the dataset’s topical focus. But the missing offensive words indicate that the hate within the Vidgen dataset might be more implicit.

**Table 3:** Words with highest PMI for each class of the selected abusive English datasets

	Words with highest PMI
Waseem - sexism	sexist, women, kat, girls, like, call, female, men, think, woman
Waseem - racism	islam, muslims, muslim, mohammed, religion, jews, prophet, isis, quran, like
Davidson - hate	bitch, faggot, like, ass, nigga, white, fuck, nigger, trash, fucking
Davidson - offensive	bitch, bitches, hoes, like, pussy, hoe, ass, got, fuck, get
Founta - abusive	fucking, fucked, like, ass, bitch, fuck, get, bad, shit, know
Founta - hateful	hate, niggas, fucking, nigga, like, people, idiot, get, amp, ass
Zampieri - OFF	liberals, like, control, gun, people, shit, antifa, get, conservatives, one
Vidgen - hostility	china, world, chinese, virus, people, ccp, us, wuhan, spread, rt

#### Overarching topic modeling

**Fig. 7:**
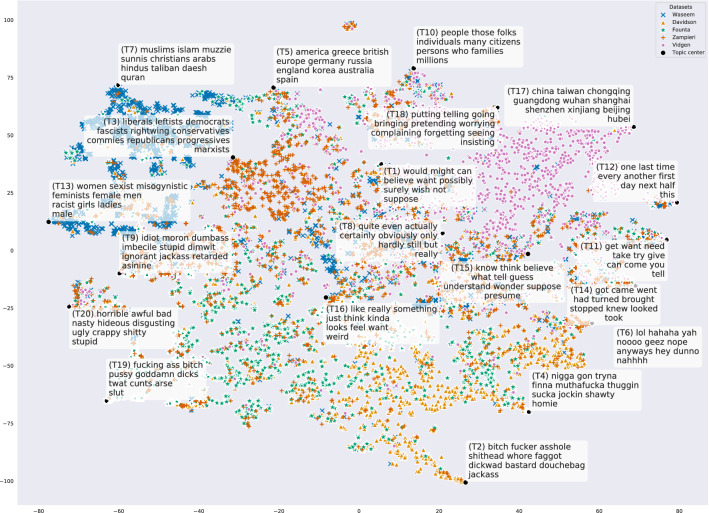
Topic model on the abusive classes of English dataset selection

Figure 7 shows the result of the topic model-based analysis on all classes. The black dots represent the centroids of the 20 identified topics. We can observe different topic biases of the datasets: A large portion of Vidgen is about viruses and China, which is not surprising due to the focus on COVID-19 (T17). Many tweets from Waseem deal with Islam. Zampieri exhibits a political focus (T3, T5: e.g., liberals, democrats, conservatives). In contrast, Davidson and Founta contain several tweets with swearwords (T2, T4, T19: e.g., bitch, asshole, nigga). These findings are in accord with the ones from the previous analysis.

### Annotation perspective

#### Distribution of inter rater reliability

**Fig. 8:**
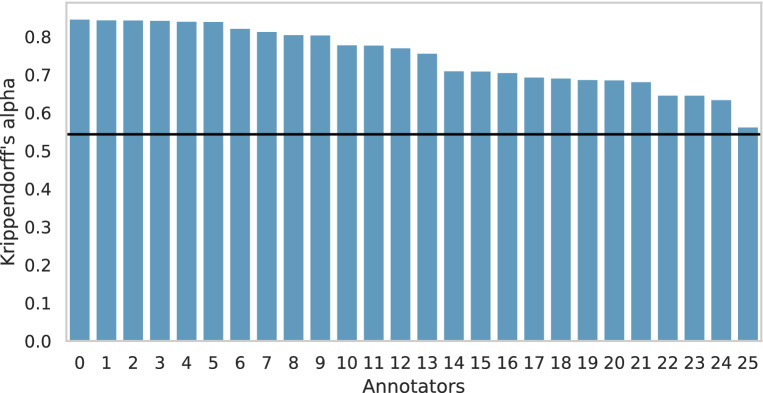
Annotators’ inter-rater reliability scores and overall inter-rater reliability score (black line) of Vidgen dataset

Unfortunately, only one dataset provides the raw annotations that are necessary for conducting this analysis. Figure 8 displays the distribution of the inter-rater reliability (Krippendorff’s alpha) of each annotator from the Vidgen dataset, sorted from highest to lowest. The horizontal line shows the overall inter-rater reliability of all annotators, where the first observation introduces the overall Krippendorff’s alpha value as 0.543. It is not an optimal value, but it is comparable to other abusive language datasets [20]. 10 of the 26 annotators achieve an individual inter-rater reality score over 0.80 between the annotators and the dataset gold standard, which is relatively good. The outlier is the last annotator with an inter-rater reliability score of 0.564. Since at least two coders annotated each document of Vidgen, one outlier cannot cause an annotator bias. Overall, the annotations of Vidgen seem to have decent quality. Based on the results of this analysis, we are not able to identify any annotator bias.

### Classification perspective

#### Cross-dataset performance

**Fig. 9:**
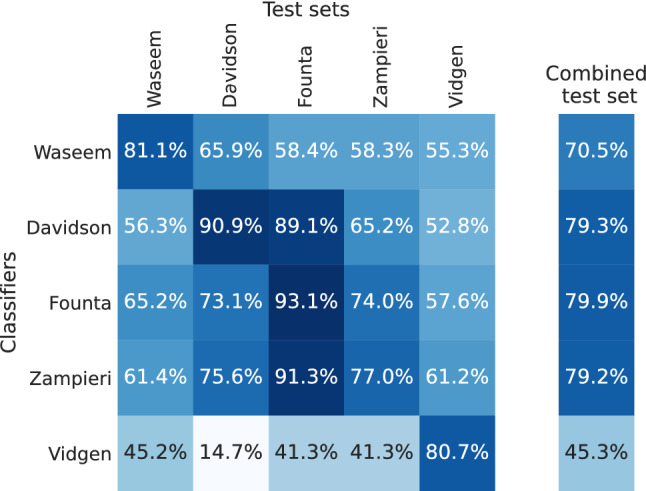
Cross-dataset classification performance (macro F1 scores)

Figure 9 presents the macro F1 scores of the classifiers that were trained on different datasets and tested on all test sets. Hate labels were unified on each dataset for the purpose of cross-classification.^3^ As the basis for the classification model, we use the English pre-trained BERT model bert-base-uncased [12].

Vidgen delivers the worst performance, but this should not surprising due to the topic focus. Davidson, Founta, and Zampieri show comparable results that are better than the ones from Waseem. Even if Davidson has the highest F1 score on the combined test set, the classifiers trained on Founta and Zampieri provide more stable results across all test sets. Therefore, these two datasets are most suitable for training generalizable classifiers.

#### Explainable classification models

**Fig. 10:**
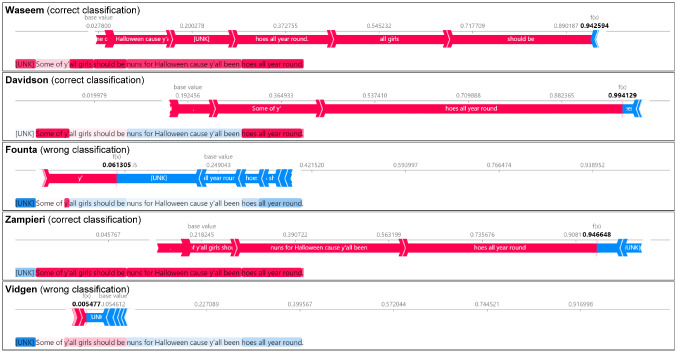
SHAP explanations of an abusive tweet that is misclassified by two of the five English classification models

Figure 10 shows the SHAP explanations for the classification of a selected tweet for each classifier. The numbers in bold represent how likely the document is classified as *abusive*. The words in red contribute to the classification as abusive, while the blue ones support the classification as *neutral*. We observe that classifiers trained on the Founta and Vidgen datasets misclassified the *abusive* tweet, while the other three correctly classified it with high confidence. In the case of Vidgen, the result should not be surprising because the dataset focuses on COVID-19-related topics and not on sexism. In contrast to that, it is unexpected that Founta seems to have a blind spot on sexism because it appears to be diverse.

## Case study 2- Arabic datasets

### Meta perspective

#### Class distribution and availability

**Fig. 11:**
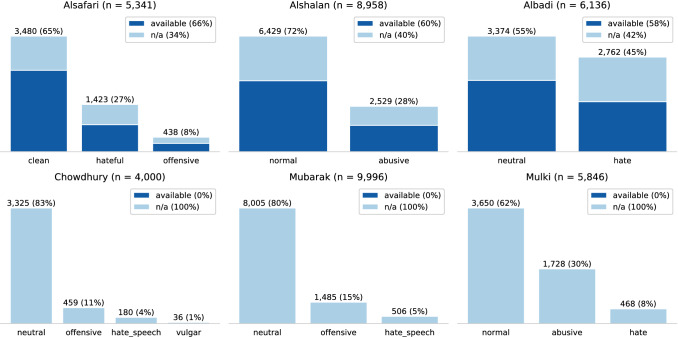
Class distribution and platform availability of Arabic datasets (*available* means that the online resource, e.g. tweet, is still accessible)

Figure 11 shows the class distributions and data availability of the Arabic datasets on the social media platforms. Similar to the English datasets, all datasets, except Mulki, are imbalanced and dominated by the neutral class. Overall, the dataset sizes are of the same magnitude and range between 4,000 and 9,996 documents. The dataset classes are more coherent than the ones from the English datasets. Regarding data availability, we can only analyze three of the six datasets because only those contain tweet IDs. We can observe a similar data degradation as for the English datasets. All classes are affected, but mainly the abusive classes. The overall range of degradation is between 34% and 42%. In the case of Albadi, we received the full dataset from the authors, which is employed for the rest of the case study. The other three datasets (Chowdhury, Mubarak, and Mulki) provide only the full text but no reference to the online resource. Therefore, we cannot consider them in the following two analysis methods.

#### Temporal distribution

**Fig. 12:**
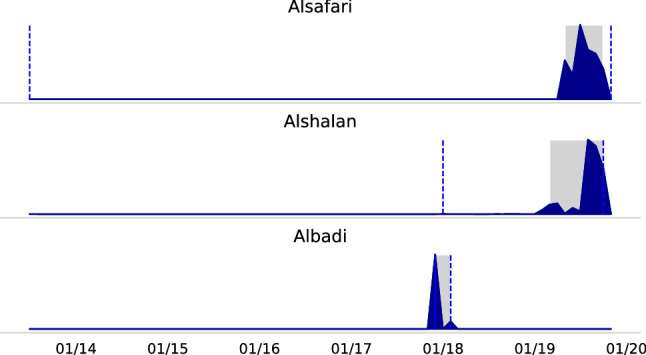
Temporal distribution of the tweets from Arabic datasets with tweet IDs

Figure 12 visualized the distribution when users posted the tweets. Overall, each dataset’s largest portions cover a short period similar to most of the English datasets. Furthermore, 95% percentile from Alsafari and Alshalan mainly come from the same period. In the context of the data degradation findings, it is surprising that the degradation rate of Albadi, which is approximately two years older than the other two, is only 2 and 8 pp higher.

#### Author distribution

**Fig. 13:**
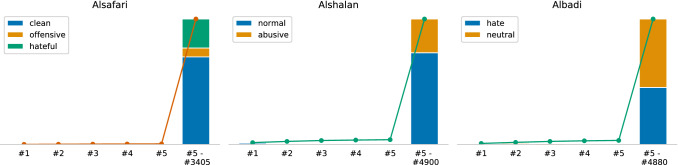
Pareto analysis showing how many tweets (incl. classes) from Arabic datasets were created by the top authors of each dataset

Figure 13 shows the Pareto analysis on the authors of the Arabic datasets that contain tweet IDs. In the case of Albadi, we received the original dataset. Thus the chart includes all authors. In contrast, the charts from Alsafari and Alshalan contain only the author data from 66%, respectively, 60% of the tweets. Overall, none of the datasets have a small group of authors that created a larger portion of the tweets, resulting in no author bias.

### Semantic perspective

#### LSI-based intra-dataset class similarity

**Fig. 14:**
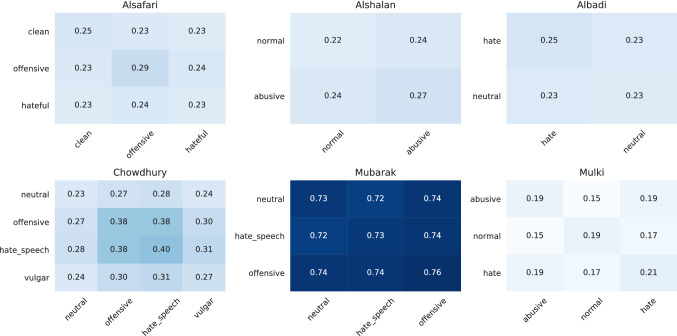
LSI-based similarity of classes within Arabic datasets (the higher the score, the more similar are two classes

Figure 14 presents the results of the LSI-based intra-dataset class similarity of the Arabic datasets. The first observation is that the LSI scores of Mubarak are higher than those of the other datasets. That means that the classes are more homogeneous by themselves than those from the other datasets. But the intra-class similarity of all classes is also in the same range. Furthermore, we can observe that the Albadi dataset is similarly homogenous. Alternatively, the *offensive* class of Alsafri and the *offensive* and *hate speech* class of Chowdhury stand out. All three classes are more homogeneous than the other classes. In the case of Chowdhury, both classes are also quite similar compared with the other two dataset classes. Based on Mulki, we can observe that the *normal* class distinguish from the *abusive* and *hate* class, while these two are similar.

#### Word embedding based inter- and intra-dataset class similarity

**Fig. 15:**
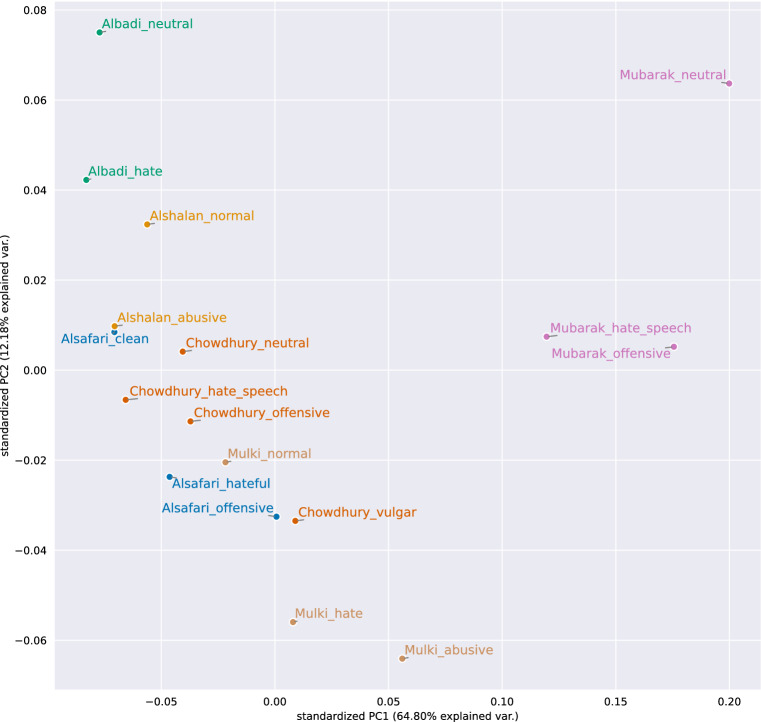
FASTTEXT sentence embedding vectors averaged for each class of Arabic datasets and visualized with PCA (the closer the points, the more similar the classes are)

For calculating the inter- and intra-dataset class similarities, we used the Arabic FASTTEXT word embeddings. Figure 15 visualizes the results. The first observation is that the abusive classes of a dataset are closer to each other than to the neutral class, which should not be surprising. But there is one exception—the *vulgar* class from Chowdhury. The Mubarak dataset is an outlier in this analysis because all its classes strongly differentiate from all others.

#### Most relevant terms of abusive classes

In Table 4, we report the words with the highest PMI for each class in each dataset. High PMI words in hateful classes differ for each dataset: while those words in Albadi’s hate class are just religious names, in Chowdhurry, they are country names, and in Mulki, they are related to Lebanese politics. The same observa-tion can be seen in the offensive and abusive class of each dataset. In Chowd-hurry, the highest PMI words in the offensive class are political, while in Mubarak, they are related to sports. The highest PMI words in the abusive class of the Alshalan dataset are not abusive, while those in Mulki’s abusive class are abusive and are also specific to the Levantine dialect.

**Table 4:**
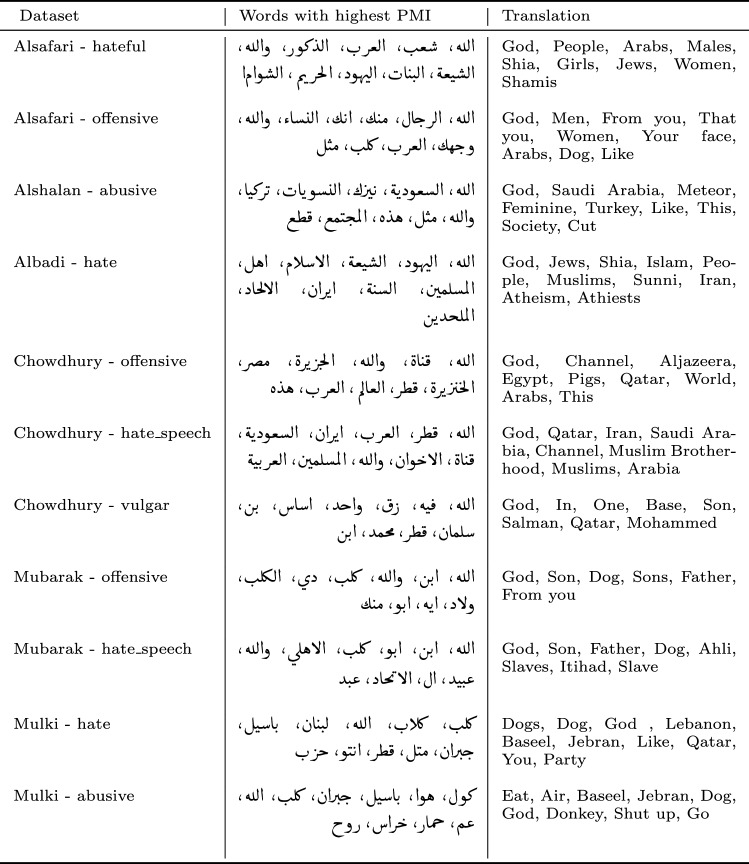
Words with highest PMI for each class of the selected abusive Arabic datasets

#### Overarching topic modeling

**Fig. 16:**
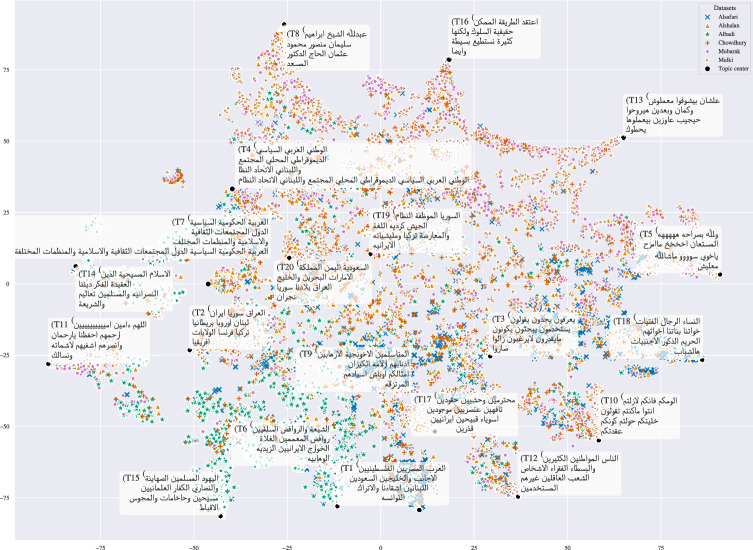
Topic model on tweets from abusive classes of Arabic datasets

Figure 16 exhibits the topic model-based analysis results on all classes. We can observe varying topic biases from the different datasets. For example, topics identified in Albadi have a religious aspect, which should not be surprising because the dataset focuses on religious hate. One of these topics is about religions, as its words contain religious names (e.g., T15 contains words such as Jews, Muslims, Christians, and Secularism). Another topic in Albadi is about different Islamic Sects (e.g., T6 contains words like Sunnis, Shia, and Salafis), and another one is about religious ideologies and doctrines (T14). Albadi seems to be the most separable dataset in terms of topics, as most of its data points fall near religious topics. Mulki and Mubarak share many topics, specifically those related to different Arabic dialects like Egyptian (T13), Levantine (T15), and standard Arabic (T10). In addition, Alsafari exhibits topics related to people from different Arabian nationalities (T1, contains words like Egyptians, Palestinians, Saudis, Lebanese) and topics related to females (T10), which is also apparent in Alshalan. Another identified topic in Alshalan is political words (e.g., T7 is reflected by words like democratic, society, union, local, and organizations).

### Annotation perspective

Unfortunately, none of the authors released the raw annotation data. Thus we are not able to conduct this analysis for the Arabic datasets.

### Classification perspective

#### Cross-dataset performance

**Fig. 17:**
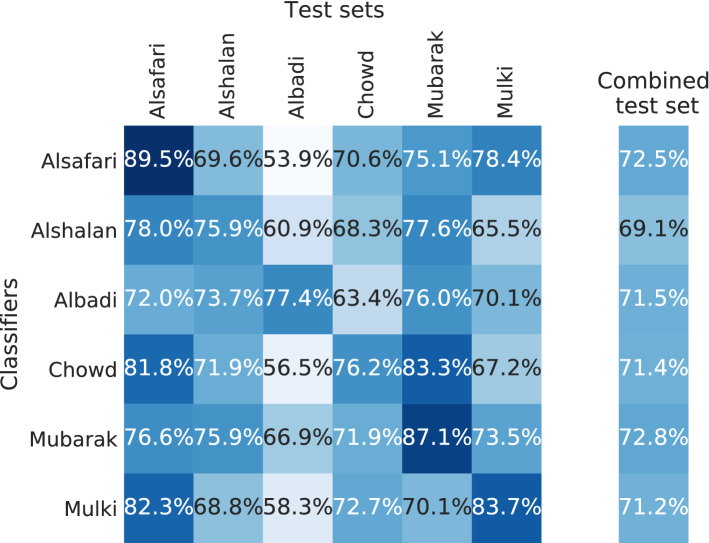
Cross-dataset classification performance (macro F1 scores) of Arabic datasets

Figure 17 presents the macro F1 scores of the classifiers that were trained on different datasets and tested on all test sets. As the basis for the classification model, we use the Arabic pre-trained BERT model asafaya/bert-base-arabic [34].

The model that performs best on its own test set is trained on the Alsafari training set. Its performance on the combined test set is slightly worse than the top classification model (Mulki). Consequently, these datasets are more suitable to train generalizable classification models. Interestingly, all classification models except the one trained on Albaldi struggle on the Albadi test set, while the Albadi classifier still provides a comparable F1 score on the combined test set. Overall, the F1 scores on the combined test set are less volatile than those from the English datasets. An explanation can be that the labeling tasks are similar, and there are no particular focuses on topics (e.g., Vidgen focuses on COVID-19-related tweets). Even if the F1 scores are lower on average than the ones from the English datasets, it is impossible to derive any conclusion from that because we use a different pre-trained model and a different number of training data.

#### Explainable classification models

**Fig. 18:**
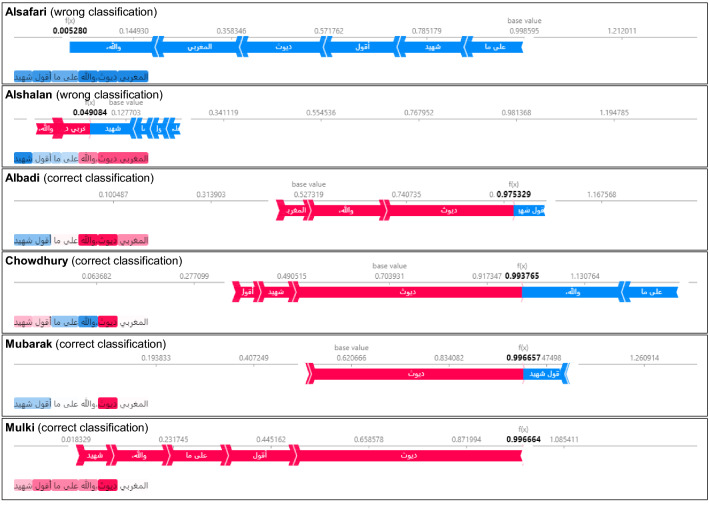
SHAP explanations of an abusive tweet that is misclassified by two of the six Arabic classification models

Figure 18 shows the SHAP explanations for the classification of a selected tweet for each classifier. The numbers in bold represent how likely the document is classified as *abusive*. The words in red contribute to the classification as abusive, while the blue ones support the classification as *neutral*.

The tweet shown in the figure translates to: *“All Moroccans are cuckolds, and God is my witness.”* This tweet is misclassified by both Alsafari’s and Alshalan’s classifier but due to different reasons. The figure shows that Alsafari’s classifier does not correlate the word *“cuckold”* with the abusive class, while Alshalan’s classifier does but the presence of the word *“witness”* (which has the same writing as the word *“martyr”* in Arabic) plays a significant role toward classifying the tweet wrongly. Other classifiers classify the tweet correctly, and for all of them, the word *“cuckold”* is the one that plays the most prominent role.

## Discussion

The results show significant differences between the compared abusive language datasets, and we identified different bias types. In both case studies, no dataset is free of common problems, and none stands out particularly positively in this regard. Each dataset comes along with advantages and disadvantages. Regarding the English datasets, Founta, for example, is a comprehensive dataset with good diversity, but it covers only a short period. Vidgen has a topic bias due to its focus on COVID-19. But such focused datasets are necessary because they address current trends. Concerning the Arabic datasets, we observed that these datasets are, on average smaller than the English ones—which could be due to resource limitations. Overall, the applied labeling schemata are more coherent than one of the English datasets. Similar to English, the datasets exhibit topical focuses (e.g., religious and political conflicts in the Middle East). Two datasets that look promising are Alsafari and Mubarak. The datasets have a decent size, the classes of both seem to be homogeneous, and they use similar labeling schemata, making them compatible. However, this has to be addressed in future work.

One may criticize that the datasets partially differentiate in the task/ labeling schema, but they all contribute to the overarching goal of fighting against abusive online language, and some are often used together in papers to evaluate classifiers. Most relevantly, all datasets are listed as abusive language classification datasets and nominally used for the same type of task.

Previous approaches to comparing abusive language data, as discussed in the Related Work section, predominantly rely on surface-level descriptive features to distinguish individual datasets. One of the main propositions of this article is that this falls short of describing the real differences of these data sets, which vary much more than can be described by these surface-level features. In light of the common challenges abusive language detection systems face, systematic bias in training data is often at the core of these issues and very hard to detect or even measure. Therefore, part of the framework’s main contribution in a structured way is to make differences in data visible on a systematic basis that goes beyond descriptive attributes and basic statistics.

In this way, the proposed framework can analyze existing datasets and relate them to other well-known datasets from the field. This is relevant for the specific analysis shown here and with other datasets used in a similar context. The focus here is not on ranking the viability of a specific dataset but on providing context for comparisons. In general, there are theoretically some aspects of the data that should be an indicator for better generalizability, such as balanced authorship or timescale and a variety of topics in both abusive and nonabusive content. However, they don’t necessarily guarantee better data distribution alone.

As this study presents a tool to evaluate and compare abusive language datasets, we are also aware of the problematic ethical circumstances of the field. The framework proposed here stems partially from the need to analyze these datasets further to discover potentially hidden problems and biases. In this role, our framework understands itself as a tool to help researchers verify the integrity of and problems present in their data and help discover potential issues with newly created datasets by comparing them to already existing data.

The system tries to be conscious of various potential biases, but it cannot claim to cover every potential bias or guarantee ethical conduct in parts of the data collection and annotation process. Therefore, researchers need to see the results in conjunction with similar methods and their efforts to guarantee data integrity. In the worst case, the framework can fail to detect bias that is not covered in the evaluation metrics. For this reason, the tool is not presented as a check to guarantee that the data is bias-free but to offer a systematic way to uncover some of the most common problems present.

Understanding the underlying data that goes into potential classification systems is an essential part of systems development. In the past, abusive language detection has shown to be easily biased against minority groups, even though this technology is meant to protect them. Therefore, we see the necessity of being conscious about the different kinds of bias within data and having a framework for analyzing and comparing them. The proposed framework’s metrics are predominantly based on either explicit metadata or evaluated in relation to the content of other datasets. This is a conscious choice to detect differences in a more fluid framework that does not rely on explicit prescriptions toward the data but should emerge from it.

Since biases can be exhibited in different ways, the methods in the framework all target various sources of conflict with the data. One of the main takeaways of the comparisons is how easy it is to separate different datasets for abusive language by some other attribute and see the dataset distinction reappear. Furthermore, these similarity measures often show more considerable variations between two different datasets than between the positive and negative classes within one of these datasets, making it clear how classifiers trained on such data might have difficulty generalizing between them.

Additionally, some of the proposed checks make it very clear what potential sources of bias might be predominant in the dataset and where to look for when assessing problems. It’s also possible to find matching datasets to “patch-up” weaknesses in a known existing one. Overall, the most important contribution of the framework lies in making potential blind spots, tendencies, and differences visible to engage with them critically in building systems.

We propose that researchers and data scientists who use different datasets to build abusive language classifiers should be aware of the datasets’ differences and biases and consider these findings during the analysis of results. So, they reduce the risk of unintended and unfair behavior of their models. Moreover, there should be increased awareness of the types of issues present in models and an incentive for dataset creators to already these parameters in mind when designing the data collection process.

Apart from general data collection issues, a few other key issues are often observed with abusive language data and become apparent when working with the datasets and using the framework.

A severe issue that we observed during our analysis is the dataset degradation that has been already mentioned by other researchers [41]. The problem is related to the procedure that some researchers publish only references/links (e.g., tweet IDs) instead of the actual text. Over time, fewer documents are available because some of them are deleted. Consequently, abusive language is deleted over time, which is good and reflects moderation efforts by social media sites. However, it impairs the reproducibility of research, reusability of data, and advances in abusive language research. This procedure also has an advantage: it preserves the user’s right to delete data. Nevertheless, we argue with respect to the degradation rate that researchers should release the text so that the datasets are persistently available. In order to address the mentioned conflict of interest between user privacy and research, we suggest anonymizing the tweets, meaning anonymous identifiers should replace the author and all usernames appearing in the text. Thus, user privacy can be preserved, and researchers can still apply our proposed framework. Furthermore, we assume that most of the tweets that are no longer available were deleted by Twitter due to policy violations. Therefore, our proposed approach should be a suitable solution.

Similarly, there is still a general problem with the ill-defined term of abusive language in general. Most often, the definition of what is considered abusive is up to the dataset creators in their labeling process. However, sometimes the criteria for the labeling is also not immediately apparent, either when done by experts or in crowdsourcing procedures. Abusive language definitions applied when labeling a dataset are therefore neither in accordance with a legal definition such as hate speech nor necessarily with the policies employed by the social media site’s data. Conversely, this is advantageous since it doesn’t tie the definition of abuse to the legal framework of one specific country or the policies of whatever social media site the content is located. However, it leads to potentially ill-defined cases where either too much, too little, or entirely different kinds of content get labeled as abusive. Therefore, it would be valuable to create very explicit datasets about the definition employed, going so far as giving examples of edge cases and how a decision was being made. In some cases, it can also make sense to define the type of task more precisely than just generalized abuse if the data is very focused on the type of content it collects.

Keeping these considerations in mind, researchers and data scientists that release new abusive language datasets should consider the following guidelines:

- They should apply our framework or a comparable one on their dataset to compare with abusive language datasets.
- The dataset should contain the full text, the mentioned metadata, and the reference to the original resource (e.g., tweet ID). To protect users’ privacy, they can anonymize the usernames in the metadata (e.g., hashing) and remove them from the full text.
- Besides an aggregated version of the annotations, they should include all raw annotations for each annotator—in the best case with metadata about the annotators.

The latter refers to annotators bias, a form of bias that we could not investigate due to missing data. Investigations into annotator bias require a great deal of transparency from the creator of a dataset, ideally encompassing descriptions of each annotator, their backgrounds, and potential biases, as well as a detailed overview of which annotator assigned what label to a particular data instance. Releasing this type of data would enable further insights into how different people annotate the same type of content and by which annotators influence some examples were labeled one way or the other. There is already research that investigates annotator bias in abusive language but requires additional data [1, 6, 45].

## Conclusions

This paper has presented an overview of a framework to describe and compare datasets from the abusive language detection domain to highlight potential problems, biases, and differences better. Therefore, we propose a multiapproach framework to investigate different aspects of the data and make them comparable beyond a discreet description framework based on labels, which has been used predominantly in the past. Our paper contributes toward helping researchers and data scientists to improve data quality and enhance their systems when collecting new data as well as when working with existing data. While our proposal was focused on the domain of abusive language detection, the proposed framework would also apply to similar NLP tasks relying on labeled data.

## Data Availability

We used only datasets that a publicly available.

## Footnotes

1 Code (including documentation and instruction for installation) available on GitHub: https://github.com/mawic/abusive-language-dataset-framework

2 For Wassem, we use the dataset provided by [27] because it contains all tweets author name.

3 Labels in bold in Table 2 are assigned to *abusive*, the others to *neutral*.

## Declarations

### Code availability

The code is published on https://github.com/mawic/abusive-language-dataset-framework.

## References

[1] Al Kuwatly, H., Wich, M., Groh, G.: Identifying and measuring annotator bias based on annotators’ demographic characteristics. In: Proceedings of the Fourth Workshop on Online Abuse and Harms, pp. 184–190. Association for Computational Linguistics, Online (2020). 10.18653/v1/2020.alw-1.21. https://www.aclweb.org/anthology/2020.alw-1.21

[2] Albadi, N., Kurdi, M., Mishra, S.: Are they our brothers? analysis and detection of religious hate speech in the Arabic Twittersphere. Proceedings of the 2018 IEEE/ACM International Conference on Advances in Social Networks Analysis and Mining, ASONAM 2018 pp. 69–76 (2018). 10.1109/ASONAM.2018.8508247

[3] Alsafari S, Sadaoui S, Mouhoub M (2020). Hate and offensive speech detection on Arabic social media. Online Soc. Netw. Media.

[4] Alshalan, R., Al-Khalifa, H.: A Deep Learning approach for automatic hate speech detection in the Saudi Twittersphere. Appl. Sci. **10**(23) (2020). 10.3390/app10238614. https://www.mdpi.com/2076-3417/10/23/8614

[5] Bender, E.M., Friedman, B.: Data statements for natural language processing: Toward mitigating system bias and enabling better science. Trans. Assoc. Computat. Linguist. **6**, 587–604 (2018) 10.1162/tacl_a_00041. https://www.aclweb.org/anthology/Q18-1041

[6] Binns R, Veale M, Van Kleek M, Shadbolt N, Ciampaglia GL, Mashhadi A, Yasseri T (2017). Like trainer, like bot? inheritance of bias in algorithmic content moderation. Social Informatics.

[7] Chowdhury, S.A., Mubarak, H., Abdelali, A., Jung, S.g., Jansen, B.J., Salminen, J.: A multi-platform arabic news comment dataset for offensive language detection pp. 6203–6212 (2020). https://www.aclweb.org/anthology/2020.lrec-1.761

[8] Church, K.W., Hanks, P.: Word association norms, mutual information, and lexicography pp. 76–83 (1989). 10.3115/981623.981633. https://www.aclweb.org/anthology/P89-1010

[9] Davidson, T., Bhattacharya, D., Weber, I.: Racial bias in hate speech and abusive language detection datasets pp. 25–35 (2019). 10.18653/v1/W19-3504. https://www.aclweb.org/anthology/W19-3504

[10] Davidson, T., Warmsley, D., Macy, M., Weber, I.: Automated hate speech detection and the problem of offensive language. Proceedings of the International AAAI Conference on Web and Social Media **11**(1) (2017). https://ojs.aaai.org/index.php/ICWSM/article/view/14955

[11] Deerwester, S., Dumais, S.T., Furnas, G.W., Landauer, T.K., Harshman, R.: Indexing by latent semantic analysis. J. Am. Soc. Inform. Sci. **41**(6), 391–407 (1990). 10.1002/(SICI)1097-4571(199009)41:63c391::AID-ASI13e3.0.CO;2-9

[12] Devlin, J., Chang, M.W., Lee, K., Toutanova, K.: BERT: Pre-training of deep bidirectional transformers for language understanding pp. 4171–4186 (2019). 10.18653/v1/N19-1423. https://www.aclweb.org/anthology/N19-1423

[13] Dixon, L., Li, J., Sorensen, J., Thain, N., Vasserman, L.: Measuring and mitigating unintended bias in text classification. In: Proceedings of the 2018 AAAI/ACM Conference on AI, Ethics, and Society, AIES ’18, p. 67–73. Association for Computing Machinery, New York, NY, USA (2018). 10.1145/3278721.3278729

[14] Fortuna, P., Nunes, S.: A survey on automatic detection of hate speech in text **51**, 4 (2018). 10.1145/3232676

[15] Fortuna, P., Soler, J., Wanner, L.: Toxic, hateful, offensive or abusive? What are we really classifying? an empirical analysis of hate speech datasets. In: Proceedings of the 12th language resources and evaluation conference, pp. 6786–6794. European Language Resources Association, Marseille, France (2020). https://www.aclweb.org/anthology/2020.lrec-1.838

[16] Founta, A., Djouvas, C., Chatzakou, D., Leontiadis, I., Blackburn, J., Stringhini, G., Vakali, A., Sirivianos, M., Kourtellis, N.: Large scale crowdsourcing and characterization of twitter abusive behavior. Proceedings of the International AAAI Conference on Web and Social Media **12**(1) (2018). https://ojs.aaai.org/index.php/ICWSM/article/view/14991

[17] Friedman B, Nissenbaum H (1996). Bias in computer systems. ACM Trans. Inf. Syst..

[18] Gebru, T., Morgenstern, J., Vecchione, B., Vaughan, J.W., Wallach, H., Daumé III, H., Crawford, K.: Datasheets for datasets. arXiv preprint arXiv:1803.09010 (2018)

[19] Krippendorff K (2004). Content Analysis: An Introduction to Its Methodology.

[20] Kurrek, J., Saleem, H.M., Ruths, D.: Towards a comprehensive taxonomy and large-scale annotated corpus for online slur usage. In: Proceedings of the Fourth Workshop on Online Abuse and Harms, pp. 138–149. Association for Computational Linguistics, Online (2020). 10.18653/v1/2020.alw-1.17. https://www.aclweb.org/anthology/2020.alw-1.17

[21] Lundberg, S.: shap.PartitionExplainer – SHAP latest documentation (2020). https://shap.readthedocs.io/en/latest/generated/shap.PartitionExplainer.html

[22] Lundberg, S.M., Lee, S.I.: A Unified Approach to interpreting model predictions. In: I. Guyon, U.V. Luxburg, S. Bengio, H. Wallach, R. Fergus, S. Vishwanathan, R. Garnett (eds.) Advances in neural information processing systems 30, pp. 4765–4774. Curran Associates, Inc. (2017). http://papers.nips.cc/paper/7062-a-unified-approach-to-interpreting-model-predictions.pdf

[23] Maaten Lvd, Hinton G (2008). Visualizing data using t-SNE. J. Mach. Learn. Res..

[24] MacAvaney S, Yao HR, Yang E, Russell K, Goharian N, Frieder O (2019). Hate speech detection: Challenges and solutions. PloS One.

[25] Madukwe, K., Gao, X., Xue, B.: In data we trust: A critical analysis of hate speech detection datasets. In: Proceedings of the Fourth Workshop on Online Abuse and Harms, pp. 150–161. Association for Computational Linguistics, Online (2020). 10.18653/v1/2020.alw-1.18. https://www.aclweb.org/anthology/2020.alw-1.18

[26] Mikolov, T., Grave, E., Bojanowski, P., Puhrsch, C., Joulin, A.: Advances in pre-training distributed word representations. In: Proceedings of the eleventh international conference on language resources and evaluation (LREC 2018). Eur. Lang. Resources Assoc. (ELRA), Miyazaki, Japan (2018). https://www.aclweb.org/anthology/L18-1008

[27] Mishra, P., Del Tredici, M., Yannakoudakis, H., Shutova, E.: author profiling for abuse detection. In: Proceedings of the 27th International Conference on Computational Linguistics, pp. 1088–1098. Association for computational linguistics, Santa Fe, New Mexico, USA (2018). https://www.aclweb.org/anthology/C18-1093

[28] Mubarak, H., Darwish, K., Magdy, W., Elsayed, T., Al-Khalifa, H.: Overview of OSACT4 arabic offensive language detection shared task. Proceedings of the 4th Workshop on open-source arabic corpora and processing tools, with a shared task on offensive language detection, 48–52 (2020). https://www.aclweb.org/anthology/2020.osact-1.7

[29] Mulki, H., Haddad, H., Bechikh Ali, C., Alshabani, H.: L-HSAB: A levantine twitter dataset for hate speech and abusive language pp. 111–118 (2019). 10.18653/v1/W19-3512. https://www.aclweb.org/anthology/W19-3512

[30] Park, J.H., Shin, J., Fung, P.: Reducing gender bias in abusive language detection. In: Proceedings of the 2018 Conference on empirical methods in natural language processing, pp. 2799–2804. Association for computational linguistics, Brussels, Belgium (2018). 10.18653/v1/D18-1302. https://www.aclweb.org/anthology/D18-1302

[31] Pearson, K.: LIII. On lines and planes of closest fit to systems of points in space The London, Edinburgh, and Dublin Philosoph. Magaz. J. Sci. **2**(11), 559–572 (1901)

[32] Poletto, F., Basile, V., Sanguinetti, M., Bosco, C., Patti, V.: Resources and benchmark corpora for hate speech detection: a systematic review. Language Resources and Evaluation pp. 1–47 (2020)

[33] Raisi, E., Huang, B.: raisi2016cyberbullying. In: Proceedings of the 2016 ICML Workshop on #Data4Good: Machine Learning in Social Good Applications. New York, NY, USA (2016)

[34] Safaya, A., Abdullatif, M., Yuret, D.: KUISAIL at SemEval-2020 Task 12: BERT-CNN for offensive speech identification in social media. In: Proceedings of the Fourteenth Workshop on Semantic Evaluation, pp. 2054–2059. International Committee for Computational Linguistics, Barcelona (online) (2020). https://www.aclweb.org/anthology/2020.semeval-1.271

[35] Salkind N (2010). Encyclopedia of research design.

[36] Sap, M., Card, D., Gabriel, S., Choi, Y., Smith, N.A.: The risk of racial bias in hate speech detection. In: Proceedings of the 57th Annual Meeting of the association for computational linguistics, pp. 1668–1678. Association for Computational Linguistics, Florence, Italy (2019). 10.18653/v1/P19-1163. https://www.aclweb.org/anthology/P19-1163

[37] Sap, M., Gabriel, S., Qin, L., Jurafsky, D., Smith, N.A., Choi, Y.: Social bias frames: Reasoning about social and power implications of language. In: Proceedings of the 58th Annual Meeting of the Association for Computational Linguistics, pp. 5477–5490. Association for Computational Linguistics, Online (2020). 10.18653/v1/2020.acl-main.486. https://www.aclweb.org/anthology/2020.acl-main.486

[38] Schmidt, A., Wiegand, M.: A Survey on hate speech detection using natural language processing. In: Proceedings of the Fifth International Workshop on Natural Language Processing for Social Media, pp. 1–10. Association for Computational Linguistics, Valencia, Spain (2017). 10.18653/v1/W17-1101. https://www.aclweb.org/anthology/W17-1101

[39] Vidgen B, Derczynski L (2021). Directions in abusive language training data, a systematic review: Garbage in, garbage out. PLOS One.

[40] Vidgen, B., Hale, S., Guest, E., Margetts, H., Broniatowski, D., Waseem, Z., Botelho, A., Hall, M., Tromble, R.: Detecting east asian prejudice on social media. In: Proceedings of the Fourth Workshop on Online Abuse and Harms, pp. 162–172. Association for Computational Linguistics, Online (2020). 10.18653/v1/2020.alw-1.19. https://www.aclweb.org/anthology/2020.alw-1.19

[41] Vidgen, B., Harris, A., Nguyen, D., Tromble, R., Hale, S., Margetts, H.: Challenges and frontiers in abusive content detection. In: Proceedings of the Third Workshop on Abusive Language Online, pp. 80–93. Association for Computational Linguistics, Florence, Italy (2019). 10.18653/v1/W19-3509. https://www.aclweb.org/anthology/W19-3509

[42] Viegas, F., Canuto, S., Gomes, C., Luiz, W., Rosa, T., Ribas, S., Rocha, L., Gonçalves, M.A.: CluWords: exploiting semantic word clustering representation for enhanced topic modeling. In: Proceedings of the Twelfth ACM International Conference on Web Search and Data Mining, pp. 753–761 (2019)

[43] Wang FK, Chen KS (2010). Applying Lean Six Sigma and TRIZ methodology in banking services. Total Quality Management.

[44] Waseem, Z., Hovy, D.: Hateful Symbols or Hateful People? Predictive Features for Hate Speech Detection on Twitter. In: Proceedings of the NAACL Student Research Workshop, pp. 88–93. Association for Computational Linguistics, San Diego, California (2016). 10.18653/v1/N16-2013. https://www.aclweb.org/anthology/N16-2013

[45] Wich, M., Al Kuwatly, H., Groh, G.: Investigating Annotator Bias with a Graph-Based Approach. In: Proceedings of the Fourth Workshop on Online Abuse and Harms, pp. 191–199. Association for Computational Linguistics, Online (2020). 10.18653/v1/2020.alw-1.22. https://www.aclweb.org/anthology/2020.alw-1.22

[46] Wich, M., Bauer, J., Groh, G.: Impact of Politically Biased Data on Hate Speech Classification. In: Proceedings of the Fourth Workshop on Online Abuse and Harms, pp. 54–64. Association for Computational Linguistics, Online (2020). 10.18653/v1/2020.alw-1.7. https://www.aclweb.org/anthology/2020.alw-1.7

[47] Wiegand, M., Ruppenhofer, J., Kleinbauer, T.: Detection of Abusive Language: the Problem of Biased Datasets. In: Proceedings of the 2019 Conference of the North American Chapter of the Association for Computational Linguistics: Human Language Technologies, Volume 1 (Long and Short Papers), pp. 602–608. Association for Computational Linguistics, Minneapolis, Minnesota (2019). 10.18653/v1/N19-1060. https://www.aclweb.org/anthology/N19-1060

[48] Zampieri, M., Malmasi, S., Nakov, P., Rosenthal, S., Farra, N., Kumar, R.: SemEval-2019 Task 6: Identifying and Categorizing Offensive Language in Social Media (OffensEval) pp. 75–86 (2019). 10.18653/v1/S19-2010. https://www.aclweb.org/anthology/S19-2010

